# Application of Programmable Tetrahedral Framework Nucleic Acid-Based Nanomaterials in Neurological Disorders: Progress and Prospects

**DOI:** 10.3389/fbioe.2021.782237

**Published:** 2021-11-26

**Authors:** Xingyu Chen, Yu Xie, Zhiqiang Liu, Yunfeng Lin

**Affiliations:** ^1^ State Key Laboratory of Oral Diseases, National Clinical Research Centre for Oral Diseases, West China Hospital of Stomatology, Sichuan University, Chengdu, China; ^2^ College of Biomedical Engineering, Sichuan University, Chengdu, China

**Keywords:** tetrahedral framework nucleic acid, tFNA, neurological disorders, DNA nanomaterials, tetrahedral DNA nanomaterials

## Abstract

Tetrahedral framework nucleic acid (tFNA), a special DNA nanodevice, is widely applied in diverse biomedical fields. Due to its high programmability, biocompatibility, tissue permeability as well as its capacity for cell proliferation and differentiation, tFNA presents a powerful tool that could overcome potential barriers in the treatment of neurological disorders. This review evaluates recent studies on the use and progress of tFNA-based nanomaterials in neurological disorders.

## Introduction

The prevalence of neurological disorders (NDs) has increased in recent years (Feigin et al., 2021). Whereas the NDs have diverse etiopathology, they share common silent progression, global incidence, and impact on life quality for patients as well as public health systems (Feigin et al., 2021).

Treatment of central nervous system disorders is a huge challenge due to the existence of the Blood Brain Barrier (BBB) and the poor understanding of the origin and pathogenesis of some neurological disorders (NDs) (Pardridge, 1998). Besides, many large functional groups in therapeutic molecules have a limited capacity for chemical modifications or the modifications adversely affect their therapeutic efficacy (Tam et al., 2020). Overcoming the challenges presented by the barriers is a priority in the development of the next generation drugs against NDs. Ideal drugs for use in the treatment of NDs should be nontoxic, biocompatible and biodegradable, be stable in blood (they should not be opsonized by the proteins), have low immunogenicity (no aggregation of platelets), have the ability to cross the BBB (particle sizes should ideally be nanoscale or they should have a receptor-mediated transport mechanism), and should be easily modified using various functional materials without adverse effects on the drugs’ therapeutic efficacy (Ramanathan et al., 2018).

In the 1980s, Nadrian Seeman reported, for the first time, that DNA could be used as a structural material which then boosted the development of DNA nanotechnology (Seeman, 1982; Seeman and Sleiman, 2017). In the past decades, various kinds of 2D and 3D DNA-based nanostructures have been reported. Due to their editable property in which the sizes and shapes could be designed properly, they all show great potentials in different biomedical fields, such as drug vehicles, biosensors and so on (Zhang et al., 2020a). But there would be a long distance from design to application of those DNA-based nanodevices and several barriers must be overcome along the way. Firstly, natural nucleic acids could not be internalized by cells because of their negative charges. However, Mao and his group found that DNA nanotubes equipped with folate acid could target receptors at the surface of cancer cells to get into the cell (Ko et al., 2008). And later, Turberfield’s group found that tetrahedral DNA nanostructures could penetrate into live cultured cells without the aid of any functional molecules, which suggested that pure DNA nanostructures of certain geometries could be internalized by mammalian cells, regardless of their surface charges (Walsh et al., 2011). And secondly, the physical environment *in vivo* is complex which requires certain resistance of the DNA structures against those changeable situations. Keum et al. compared the stability between tetrahedral nanostructures and the linear DNA strands. And as revealed by electrophoresis, the tetrahedral structure was much more stable in the presence of specific and non-specific nucleases than linear strands (Keum and Bermudez, 2009). Besides, most functional biological molecules are fragile and may only be functional in aqueous environments which demand vehicles of certain 3D geometries and nanometer-sized features. Tetrahedron, as one of the simplest nanostructures for DNA nanotechnology, has attracted much attention. Fan successfully, demonstrated that the tFNA was compact, mechanically stable, nontoxic, and resistant to nuclease degradation (Li et al., 2019). And based on Watson-Crick pairing rules, this simple tetrahedron structure could be well designed into different sizes and modified with predictable loading sites for functional molecules. Moreover, most nanostructures are mainly used as vehicles to transfer functional molecules to the target with less unwanted waste and side effect while tFNA itself has positive effects on particular cells and even systems which would be discussed below.

Recent evidence suggests that tFNA, a special DNA nanodevice, which has been widely used in diverse biomedical fields, might be a powerful tool that could be helpful in the treatment of NDs (Li et al., 2021b; Li et al., 2021c). Here, this minireview concludes the development and use of tFNA, which is highly applied DNA nanostructure with unique characteristics, flexible penetration, precise programmability and susceptibility to biological regulation (Sirong et al., 2020; Sun et al., 2021). Besides, we highlight the application of the tFNA in NDs and current challenges as well as potential prospects.

## Tetrahedral DNA Nanostructures

### General Characteristics

**FIGURE 1:**
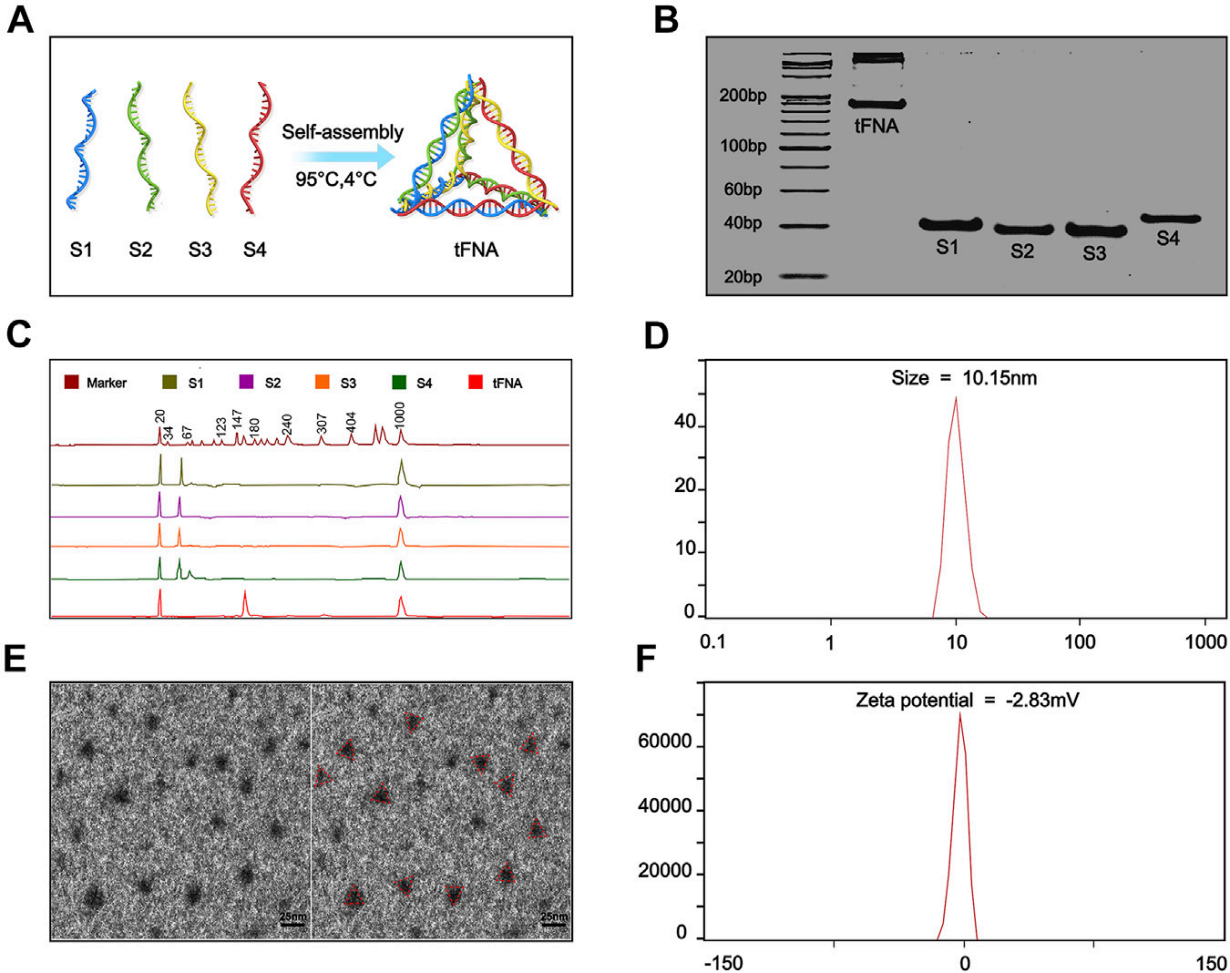
General characteristics of tFNA. **(A)** Synthesis of tFNA. **(B)** The successfully synthesis of tFNA confirmed by 8% PAGE. **(C)** The molecular weight of ssDNA and tFNA detected by high performance capillary electrophoresis. **(D)** The size of tFNA. **(E)** The size and distribution of tFNA analyzed by transmission electron microscope (red triangle). Scale bars are 25 nm. **(F)** The Zeta potential of tFNA. This article was published in Applied Materials Today, vol.24, Yuting Yang, The remyelination effect of DNA framework nucleic acids on demyelinating diseases, Page No.1-3, Copyright Elsevier.

TFNA, first synthesized by Goodman in 2004, are characterized by a tetrahedral morphology (Goodman et al., 2004). The commonly used edge length of the tFNA is about 20 bp and is ∼10 nm in size as detected by DLS with a slightly negative charge in zeta potential (Figure 1).

Notably, Fan and colleagues designed a double stranded DNA (dsDNA) with variable edge lengths (37, 26, 17, 13 bp, or 7 bp) to achieve different sizes (Lin et al., 2016). The commonly used DNA tetrahedron comprises of four strand DNAs whose edge lengths are 63 nt, and 50% GC content in every strand (Goodman et al., 2004). Consequently, based on Watson−Crick base pairing protocol, the four single stand DNAs complement each other, fold into 3 blocks with 20 bp on each side, which leaves a single nucleotide at every vertex for flexibility (Peng et al., 2016).

### Membrane Permeability

Due to its negative charges, DNA does not directly cross the plasma membrane to enter cells. In this regard, Liang et al. showed that tFNA can enter cells through caveolin-mediated endocytosis and be translocated into lysosomes in a microtubule-dependent manner, implying structural stabilities. And he also reported that tFNAs could enter into the cells with corner by reducing the influence of electrostatic force and the dynamic process may result in the charge redistribution of the mobile cell membrane (Liang et al., 2014). Later in 2019 with advanced force tracing technique which could detect the process down to pico-newton and milliseconds, Chen noticed slight rotation of the tFNAs during the transmembrane process, which implied that the orientation adjustment of tFNAs might account for the difference in cell entry. And also, he found that the tFNAs with different size exhibited different rotation angles in the final stage. Besides that, Chen found that the transportation of tFNA in transmembrane was depended on caveolin-mediated endocytosis, which was consistent with Liang’s report (Chen et al., 2020). Fan also expanded its application. He tested several distinct shaped DNA structures on the field of transdermal drug delivery (Wiraja et al., 2019). It was interesting that the transdermal ability of DNA structure was more dependent on size instead of shape, among which tFNA with 21 bps could reach the deepest place. Of course, cell membrane permeability is not unique to FNAs. Nanomaterials with specific chemical properties and desirable sizes and shapes like gold-based nanomaterials could still achieve sound membrane permeability (Wang et al., 2018). With the mysteries of cell membrane gradually being uncovered, the measures to better the permeability of nanomaterials would be figured out.

### Programmability

High DNA tetrahedron programmability determines its ability to undergo design combinations with various materials to overcome its usability challenges in life science. Because of its high biocompatibility in cells, its ability to deliver drugs into the targeted site and boost their efficacy has been reported (Zhang et al., 2020a).

However, application of this nanodevice on cell culture medium is not enough and requires evaluation in physiological environment *in vivo*. For example, *in vivo* divalent cation concentrations, such as Ca^2+^ or Mg^2+^, are below ideal ranges for maintenance of structural integrity (Douglas et al., 2009; Ponnuswamy et al., 2017). Besides, the metabolic effects as well as plasma proteins in body fluids have an effect on the half-life of tFNA and its durability (Conway et al., 2013). Moreover, most of the tFNA are digested by intracellular lysosomes, which negatively impacts their efficacy (Liang et al., 2014; Halley et al., 2016).

**FIGURE 2:**
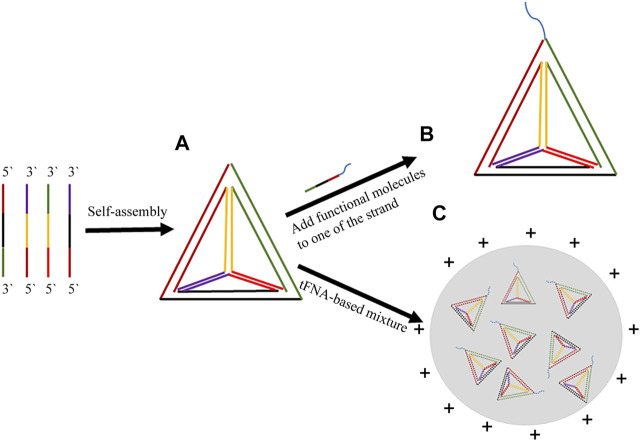
Synthesis and programmability of tFNA-based nanomaterials. **(A)** Synthesis of tFNA with four strands. **(B)** Simply adding functional groups such as aptamer, cell-penetrating peptides, et al. to tFNA; **(C)** TFNA-based mixture.

There have been efforts to improve tFNA and prolong its sustainability both in the cell and in the circulatory system. The most common way involves the attachment of functional molecules to the strands to reduce unnecessary wastage as well as adverse side-effects before the tFNA-based nanoparticles arrive at the target sites (Figure 2).

Aptamers are the common molecules used to help on the modifications since they can form three dimensional structures that specifically bind to targets that range from small molecules to whole cells (Zhang et al., 2020a). Previous data showed high compatibility of the tFNA with aptamers. For instance, Tian. et al. added AS1411 to one of the four strands and synthesized an aptamer-modified tFNA (Apt-tFNA) (Tian et al., 2020). Then, under hypoxia, they contrasted intracellular localizations of AS1411-modified tFNA with those of unmodified tFNA in various cells. Besides, under hypoxic conditions, they assessed the effects of tFNA and Apt-tFNA on cell proliferation and cell cycle. The data demonstrated that the Apt-tFNA are efficient tumor-targeted drug delivery vehicles compared to the tFNA, and they can suppress tumor cell proliferation. In addition, other targeting moieties, such as folic acid, cell-penetrating peptides and affibody molecules have been used to modify the tFNA to improve the specificity (Xia et al., 2016; Zhang et al., 2017a; Zhang et al., 2017b; Zhang et al., 2020b; Meng et al., 2021).

Besides the simple addition of functional groups to the strand, there have been efforts to design systems that protect tFNA or design a step-by-step releasing system (Figure 2). Ge. et al. used positively charged PEGylated protamine with the tFNA and the data showed an increase in endocytosis as well as longer survival in biological fluids (Ge et al., 2019). On the other hand, Xue. et al. designed a fusion protein with tFNA to enhance the uptake of the tFNA in cancer cells as well as longer retention time in circulatory system (Xie et al., 2018). In addition, Yan. et al. applied a complexed tFNA-based system to overcome multidrug resistant cancer (Yan et al., 2021). The system could “poke holes” on cell membranes and enhance cellular internalization as well as bypass endocytosis regardless of drug-resistant mechanisms.

### Biological Regulatory Ability to Various Cell Types

Recent data has shown that the tFNA could regulate cellular behavior, with different effects on various cell types (Zhang et al., 2021; Zhang et al., 2022). Shi's study reported increased autophagy in chondrocytes following exposure to tFNA (250 × 10^–9^ M) (Shi et al. 2017). On the other hand, Zhou. et al. applied tFNA to human periodontal ligament stem cells and the data demonstrated its positive effect on proliferation and osteogenic differentiation (Zhou et al., 2021). Ma. et al. assessed the effects of tFNA on neural stem cells (NSCs), on self-renewal as well as differentiation of neuroectodermal stem cells and showed that the tFNA could positively promote their proliferation and differentiation (Ma et al., 2018a). Thereafter, several studies have been conducted on cell models of different diseases such as skin lesions (Zhu et al., 2020).

Based on the above properties that might be potentially used to overcome the challenges that exist in neurological systems, the tFNA is a potential tool in development of promising therapeutic drugs. The advantages and disadvantages of tFNA-based nanomaterials used in biomedical fields are summarized in Table 1. Recent studies have attempted to apply tFNA-based nanomaterials in the improvement of various neurological disease models and some have shown promising outcomes.

**TABLE 1:** The advantages and disadvantages of tFNA-based nanomaterials used in biomedical fields.

Advantages	Disadvantages
①Enhanced tissue-penetrating and cellular uptake ability without extra molecules	①Little research about the specific mechanisms of organ’s selective uptake and interaction with various cells and tissues
②Greater resistance to biological environments	②No systematic studies about long-term effects on biologism
③Exclusion of viral DNA sequences from preparation and application	③Little research about the pharmacokinetics
④Facile preparation in high yields	
⑤More than a vehicle	

## Application of TFNA-Based Nanomaterials in Neurological Disorders

### Peripheral Nervous System Diseases

#### Facial Nerve Injury Repair

In the maxillofacial areas, facial nerves are crucial, and their injury might result into depressed auditory and gustatory sensory losses, motor functions of facial muscles, dysregulated salivary gland secretion or limb paralysis, which affects the mental, physical, and social health of patients (Yao et al., 2021). Regrettably, promising drugs, such as steroids, vitamin B12 (VB12) exhibit incomplete, gustatory and inadequate clinical outcomes by failing to achieve complete injured nerve restoration. Hence, alternative strategies that focus on modulation of Schwann cells are often breaking point of neurorestoration as Schwann cells play an important role in the process of facial nerve repairing (Jessen et al., 2015).

For instance, Yao’s study incubated 125 × 10^–9^ M tFNA for 24 h in the presence of Schwann cells, and showed that tFNA could enhance cell proliferation, migration, secretion of functional proteins *via* activation of the neuroprotective signaling pathway (NGF/PI3K/AKT) in the Schwann cells (Yao et al., 2021). In facial nerve crush models, the tFNA promoted the recovery of nerve conduction, muscle movement, repair of injured myelin sheaths and axons as well as expression of various marker proteins like neurotrophic factor, myelin basic protein (MBP), nerve growth factor, and peripheral myelin protein 22. These marker proteins could promote injured neuron repair and formation of mature myelin sheaths during the remyelination process. Thus, the data robustly demonstrated the successful application of the tFNA in the restoration of injured facial nerve.

#### Retinal Ischemia-Reperfusion Injuries

Retinal ischemia-reperfusion injuries occur during the pathological processes of various ophthalmic diseases, such as diabetic retinopathy, glaucoma or retinal arterial occlusion (Cho et al., 2015; Coucha et al., 2015; Tanito et al., 2016). Ischemia-reperfusion injuries are accompanied by high reactive oxygen species (ROS) levels, which cause retinal ganglion cell (RGC) damage, fuel their apoptosis and may result in irreversible visual field loss (Cho et al., 2015). However, as terminally differentiated cells, RGCs are incapable of regenerating and there is fewer effective drug in the treatment of loss of visual field because of oxidation and apoptosis of the RGCs (Cassereau et al., 2011; Sugiyama et al., 2009; Tanito et al., 2016). Therefore, there is a great need for alternative strategies that focus on antioxidants to achieve neuroprotective goals to support and complement existing ophthalmic therapeutic approaches (Pardue and Allen, 2018).

Qin used tert-butyl peroxide to developed RGC models of oxidative stress (Qin et al., 2019). Their findings demonstrated that tFNA suppressed cellular ROS levels and protected the cells from oxidative stress through intracellular oxidation-associated enzyme regulation via akt/nrf2 signaling pathway activation. Moreover, Li synthesized a novel DNA nanocomposite (tFNA-miR22) by linking microRNA-22-3p (miR-22) to tFNA (Li et al., 2021a). The results showed that tFNA can effectively transfer miR-22 to damaged RGCs and have a neuroprotective effect on glaucoma. Besides, they found a certain synergy effects between tFNA and miR-22. TFNA-miR22 can selectively activate tyrosine kinase receptor B to increase the expression level of brain-derived growth factor (BDNF), thereby exerting a neuroprotective effect on RGCs. This study established a simple and effective micro-RNA delivery system, which might be a promising neuroprotective agent for the treatment of optic nerve degenerative disease in the future.

### Spinal Cord Injury

Spinal cord injury (SCI) is a damage to the spinal cord and characterized by axon disruptions and irreversible neuron loss, leading to dramatic disability in patients. Neuroprotection as well as neuroregeneration are two current strategies in the treatment of SCI. Nerve regeneration treatments, such as cell transplantation and tissue engineering, aim to reconstruct the connections of damaged neuronal tissues through recellularization of the spinal cord injury site, promote the regeneration of axons and neurons, and restore nerve loss.

The purpose of treatment for spinal cord injury is to reconstruct the connections of damaged neuronal tissues through the recellularization in the injured site of spinal cord, thereby promoting the regeneration of axons and neurons and restoring neural loss (Mahar and Cavalli, 2018; Wang et al., 2019). Although some therapeutics have been verified with effects on SCI in previous studies, their applications are still limited by the low efficiency and high cost *in vivo* (Wang et al., 2019; Zhang et al., 2019). Therefore, a novel method of neuroregeneration and neuroprotection combined with cost-efficiency and high biocompatibility for the treatment of SCI might be a promising new therapy.

TFNA has been shown to promote the proliferation, migration, as well as differentiation of NSCs into neurons (Ma et al., 2018a; Ma et al., 2018b). Ma showed that simultaneous treatment of NSCs using tFNA increased the survival of transplanted NSCs, thus promoting tissue regeneration in lesion sites and achieving the recovery of motor function as demonstrated by Basso-Beattie-Bresnahan score (Wu et al., 2020; Ma et al., 2020). In addition, the study showed that tFNA could reestablish myelin sheaths by upregulating the expression levels of the MBP protein, a marker protein in oligodendrocytes. This study provided a new choice with high biocompatibility and cost-efficiency for the improvement of SCI.

### Cerebrovascular Disease

#### Intracranial Hemorrhage (ICH)

Intracranial hemorrhage (ICH) is a common cerebrovascular disease caused by primary conditions including cerebral aneurysm, moyamoya disease, arteriovenous malformation, traumatic brain injury, hypertension and amyloid angiopathy, and is associated with poor prognosis and high mortality (Keep et al., 2012). During the acute ICH phase, C-C chemokine receptor 2 (CCR2) plays a central role in the recruitment of inflammatory/immune cells, followed by polarization of most of the microglia towards the M1 (pro-inflammatory) phenotype and expression of various inflammatory molecules, such as IL-1β^39^, TNF-α^40^, IL-6^41^, CX3CL1^42^as well as iNOS^43^ which enhances neuronal apoptosis, nerve conduction bundle fracture as well as BBB disruption (Charo and Ransohoff, 2006; Liesz et al., 2011; Sansing et al., 2011; Yang et al., 2013; Matsushita et al., 2014; Zhang et al., 2014). Thus, effective regulation of microglia polarization and activation during neuroinflammation is the focus of various neurological studies on secondary brain injury after ICH. Therefore, inhibition of CCR2 expression might provide new therapeutic strategies for ICH-specific secondary brain damage.

On the other hand, Fu developed a DNA nano-drug referred to as tFNA-siCCR2 consisting of a siCCR2 attached to a distinctive functional tFNA (Fu et al., 2021). They showed that 250 × 10^–9^ M tFNA-siCCR2 could significantly attenuate the CCR2 gene expression, and promote a phenotypic M1 to M2 switch *in vitro*. Besides, they showed that after intraventricular injection, tFNA-siCCR2 could bypass the BBB, avoid immune clearance and inhibit neuroinflammation in an ICH mouse model. Moreover, the treatment could accelerate hematoma absorption and partially preserve motor nerve functions by inhibiting the release of inflammatory molecules like IL-1β, iNOS, TNF-α, and IL-6 as well as upregulate various anti-inflammatory factors, including CD36, Nrf-2 and IL-10. Thus, applications of tFNA as a vehicle of siCCR2 in the treatment of ICH through the regulation of microglia presents an improved therapeutic strategy with a clinical translation potential.

### Neurodegenerative Diseases

#### Alzheimer’s Disease

Alzheimer’s disease (AD) is a prevalent progressive neurodegenerative disorder that is associated with mild cognitive impairment and neuronal damage. This disease has the potential to evolve into dementia (Shah et al., 2016; Ayala and Colanzi, 2017). The pathomechanisms of AD are not yet clearly understood, but a widely accepted etiology associates it with the deposition of Aβ proteins in the brain, which triggers various pathological processes such as cell cytotoxicity and mitochondrial dependent cell apoptosis (Wisniewski et al., 1997; Zheng et al., 2002; Esteban, 2004). Therefore, a number of studies are evaluating biocompatible methods that could be used in the clearance of the Aβ proteins in the brain (Han et al., 2016; Luo et al., 2016).

An *in vitro* study by Shao showed that 250 × 10^–9^ M tFNA for 6 h could protect as well as rescue PC12 cell death via Aβ-mediated PC12 cell apoptosis by modulating abnormal cell cycles, restoring dysregulated nuclear morphologies and caspase activities. In addition, the tFNA led to significant suppression of the ROS levels and expressions of cell apoptosis-associated proteins like caspase 3 and bax through ERK1/2 pathway activation (Shao et al., 2018).

In addition, *in vitro* as well as *in vivo* experiments by Shao further demonstrated that 250 × 10^–9^ M tFNA could effectively inhibit apoptosis by influencing the expressions of various apoptosis-related proteins like caspase 3, bcl2 and bax in an AD cell model. Moreover, it improved memory and learning abilities in rat models of the AD (Shao et al., 2020). In Nissl-stained sections and tunnel-stained sections of the hippocampus of a rat model, tFNA injections into tail veins markedly suppressed Aβ25-35 expression and inhibited AD-induced apoptosis in the hippocampus. These results imply that tFNA can be used as new therapeutic options for AD by reversing the deposition of Aβ25-35 protein-induced neuronal loss.

#### Parkinson’s Disease

Parkinson’s disease (PD), a neurodegenerative disorder, is characterized by a series of basal ganglia dysfunction, decreased dopaminergic neurons in the substantia nigra, abnormal Lewy body and neurite accumulation as well as progressive motor disorders (Wu et al., 2020). Current anti-parkinsonian agents like dopamine or surgical deep brain electrical stimulation exhibit unsatisfactory effects on the control of PD (Charvin et al., 2018). Therefore, there is a need to develop effective drugs for PD that can improve motor symptoms and inhibit the accumulation of misfolded α-synuclein.

1-methyl-4-phenyl-1,2,3,6-tetrahydropyridine (MPTP) and its metabolite induced apoptosis in PC12 cells via suppressing α-synuclein, a PD biomarker. To evaluate the protective as well as restorative effects of tFNA on neurotoxicity, Cui treated the PC12 cells using MPTP and/or tFNA (Cui et al., 2019). The findings showed that tFNA inhibited MPTP-induced apoptosis of PC12 cells *via* suppression of the α-synuclein biomarker. The gene and protein expression analysis further demonstrated that the tFNA exhibit neuroprotective as well as neurorestorative effects on the PC12 cells by upregulating Pi3k/akt pathway and fueling apoptosis related proteins such as caspase 3, bcl2 and bax. This study highlighted the therapeutic potential of the tFNA in reducing the neuropathological changes caused by PD.

Another study reported that through electrostatic incubation, the tFNA could successfully transport therapeutic VB12 across the BBB in the treatment of PD (Cui et al., 2021). VB12 is a potential therapy for PD that has been shown to promote autophagy in PD models. However, its therapeutic outcomes are limited by its dependence on transporters as well as its exceptionally low brain tissue utilization. Synthesis of VB12-loaded tetrahedral framework nucleic acid (TVC) led to increased BBB-penetration characterized by inhibition of LRRK2 protein kinase and restoration of autophagy of striatum, thus increasing the time spent on pole tests of a MPTP-induced PD mice model. Although there are still challenges such as purification of TVC and the structural basis of interactions between TVC and LRRK2 that need further evaluation, these novel therapeutic methods could enable the tFNA be used as a potential drug carrier in the treatment of PD and other NDs.

### Demyelinating Disease

#### Multiple Sclerosis

Multiple sclerosis (MS) is one of the most famous and common demyelinating diseases in young adults (Nocentini et al., 2012). MS is characterized by multifocal demyelinating lesions, inflammatory infiltrates, axonal damage as well as deletion of oligodendrocytes (Friese et al., 2014). There are no first-line drugs against MS and thus any progress achieved in remyelinating field would be helpful to delay disease progression (Way et al., 2015).

Yang showed that, through regulation of mitochondrial apoptotic related proteins such as caspase 3, bcl2 and bax, 250 × 10^–9^ M tFNA rescued oligodendroglia progenitor cells from interferon-γ mediated cell death *in vitro* (Yang et al., 2021). Subsequent *in vivo* experiments used cuprizone diet for demyelination to establish a mouse model of MS. The study showed that tFNA enhanced remyelination and enrichment of myelinated axons by promoting the expression levels of myelin-related proteins such as MBP and suppression of apoptosis in the corpus callosum region. The study highlighted the therapeutic significance of tFNA for MS.

### Tumors in Central Nervous System

#### Glioblastoma Multiforme

Glioblastoma multiforme (GBM) is a frequent and aggressive primary human brain tumor that is correlated with high mortality rates as well as poor prognosis. This disease is characterized by a survival time of <18 months after diagnosis (Stupp et al., 2005; Hoffman et al., 2019; Kumar et al., 2019). Hemotherapy is considered to be an essential auxiliary treatment after surgery. However, the BBB and blood–brain tumor barrier prevent drug delivery into the glioma tissues, coupled with the nonspecific and nontargeted nature of current drugs present major challenges.

Shi bind Gint4. T (aptamer that binds and inhibits PDGFRβ, the hallmark of GBM) and GMT8 (binds specifically to U87MG glioma cells) to ssDNA2 (S2) and ssDNA 3 (S3) into Gint4. T-tFNA-GMT8 (GTG) (Shi et al., 2019). They showed that GTG could target and inhibit the proliferation of glioma cells and help ferry anti-tumor drug paclitaxel cross the BBB. Besides, the GTG could induce apoptosis in U87MG cells when loaded with paclitaxel. In addition, a combination of paclitaxel and GTG has great potential for glioma therapy and tFNA can significantly aid in drug delivery.

On the other hand (TMZ) has been used in GBM treatment. However, effective GBM therapy is threatened by resistance and suppression of the bone marrow. Fu synthesized a tFNA nanoparticle that could load TMZ and modified the tFNA using GS24, a DNA aptamer which could bind transferrin receptors in cerebral vascular endothelial cells of mice thereby enabling the tFNA nanoparticle to cross the BBB (Fu et al., 2019). The study showed that, after incubation of the TMZ with TFNA-GS24 for 6h, TFNA-TMZ could cross the BBB and yield a significant effect in destruction of TMZ-sensitive cells (U87 and A172) compared to a single TMZ agent. Moreover, the tFNA-TMZ attenuated drug resistance in the TMZ-resistant cells (LN-18 and T98G) via downregulation of O6-methylguanine-DNA-methyltransferase. These results further confirmed that tFNA-TMZ is an effective nanoscale carrier in therapeutic drug delivery across the BBB and thus could enhance the efficacy of drugs against glioblastoma.

### The Possible Mechanisms of TFNA-Based Nanomaterials Used in Neurological Disorders

**FIGURE 3:**
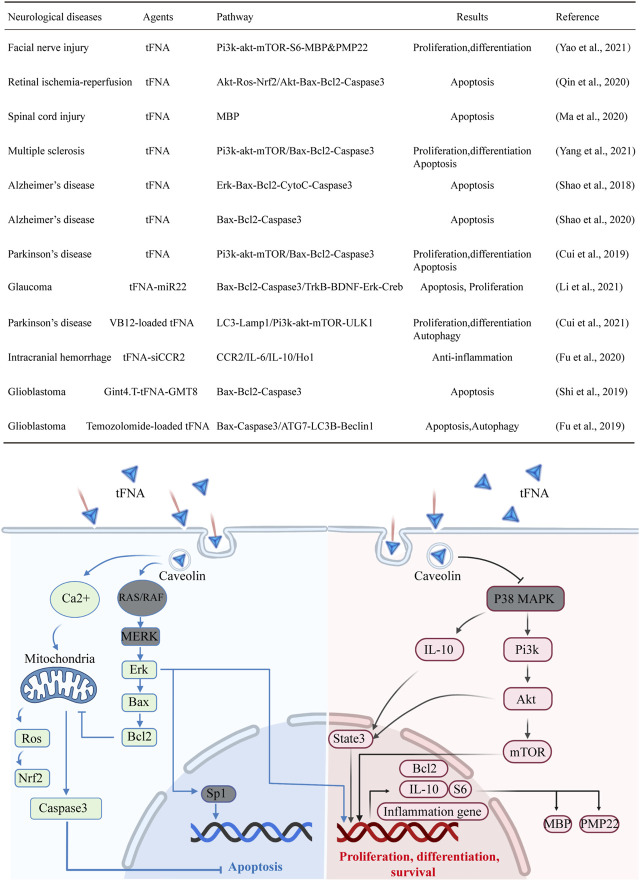
The possible mechanisms of tFNA-based nanomaterials used in NDs.

Overall, the application of programmable tFNA-based nanomaterials in NDs could be divided into two categories. The first type is simplex tFNA, which utilizes its own biological effects and the second type is tFNA complex, which is carried with pharmacological agents like VB12 and/or modified with targeting functional groups like GMT8 to play synergistic roles. The underlying mechanisms of simplex tFNA to treat NDs are mainly related to the regulations of two signaling pathways: 1) The mitochondrial apoptosis pathway represented by bcl2-bax-casepase3 components. 2) The cell proliferation and differentiation pathway represented by pi3k-akt-mTOR components. Through promoting neuronal proliferation and differentiation and inhibiting neuronal apoptosis to supplement neural loss and/or restore neural function, tFNA plays positive biological effects in NDs like peripheral or spinal cord nerve injury and neurodegenerative diseases (AD, PD, MS). When it comes to tFNA complex used in NDs, the mechanisms change according to the loaded drugs and the modifying groups. For example, in the treatment of PD by VB12-loaded tFNA complex, beside promoting neuronal proliferation by tFNA, the VB12 contributes to autophagosome formation by promoting LC3-Lamp1 interaction; and in the treatment of intracranial hemorrhage by tFNA-siCCR2 complex, the siRNA CCR2 silenced the expression of CCR2 to play anti-inflammation roles. To be better clarified, we concluded the signaling pathway of tFNA-based nanomaterials influenced in NDs in table and figure forms **(** Figure 3 **)**.

With those positive *in vitro* and *in vivo* outcomes, it is not difficult to expect the further development for this smart nanomaterial. However, it is also clear that those studies are still in very early stages for this material to go into the clinical practice. Puzzles must be answered properly before tFNA could be identified as one outstanding candidate for the treatment of NDs.

• Most importantly, the underlying mechanisms of tFNA’s multifunctional effects are poorly studied. Though this article focuses on the recent research outcome of tFNA in neurological system, more further researches are still ongoing to unseal the secrets. Here we address some questions that urgently needed to be solved. Could the bcl2-bax-casepase3 or pi3k-akt-mTOR related signaling pathway be the final answer? How could tFNA influence these signaling pathway? The perfect balance among those systems is the key point for our body to function in a harmonious way. So, could it be the outcome of the interaction among other systems? The question listed here is from a recent data that tFNA itself had the potential ability of immunomodulatory (Gao et al., 2021). Therefore, there is a thrilling direction for us to dig in and also the next goal to achieve.
• As mentioned before, treatment of central nervous system disorders is a huge challenge attributed to the existence of BBB. In Fu’s study, they have observed that TFNA-TMZ could cross the BBB by two-photon microscopy (Fu et al., 2019). However, like other studies above, the mechanism of how tFNA-based nanomaterials crossing the BBB is not well studied. Besides, the underlying mechanism of tFNA penetrating into the cells has not been fully understood too.
• Biosafety cannot be simply ignored even if the material DNA is biodegradable and biocompatible. Before we put these DNA nanostructures into clinical practice, their potential immunostimulatory properties must be systematically investigated. Although many of the animal studies reported good biosafety of tFNA-based nanomaterials by collecting H&E staining data of important organs like heart, liver, spleen, lung and kidney. Another important question whether tFNA-based nanomaterials or their metabolites could be aggregated and exert toxicity in the brain is not clear. According to a recent study, tFNA exhibited immunomodulatory capability in decreasing the percentage of Th1 cell subgroup and inducing Treg differentiation (Gao et al., 2021). But there are still several critical issues to be studied. For example, how do the physiochemical properties of DNA nanostructures affect the whole circulatory system, would those foreign DNA sequences have prolonged negative effect on genetics or where is the safe line for those DNA sequences and what is the final fate for those materials?
• Structural stability is another significant foundation for robust application of tFNA in NDs. In systemic delivery, DNA nanostructures are highly sensitive to fluctuation of temperature, cation concentration and ribonuclease. For example, when applied *in vivo*, the relatively lower cation concentration and existence of ribonuclease always contribute to structure disintegration (Ramakrishnan et al., 2018). Besides, the ribonucleic acid nature determined poor thermal stability has restricted tFNA uses on photothermal therapies though the poor thermal stability might be utilized in temperature-responsive systems. Recently published papers reported that coating tFNA-based nanomaterials with protective agents such as PEGylated protamine increases its stability and some studies reported surprisingly increasing stability of DNA origami adsorbed on solid substrates such as mica flakes (Ge, et al., 2019; Ramakrishnan et al., 2018). However, further experimental and theoretical studies on molecular mechanisms involved in thermal stability still needed before clinical application of tFNA-based nanomaterials in NDs or in other diseases.
• Pharmacokinetics (*in vivo* circulation, distribution, metabolism, etc.) of this DNA nanostructure make sense too. However, the available studies are far from comprehensive.

## Conclusion

**FIGURE 4:**
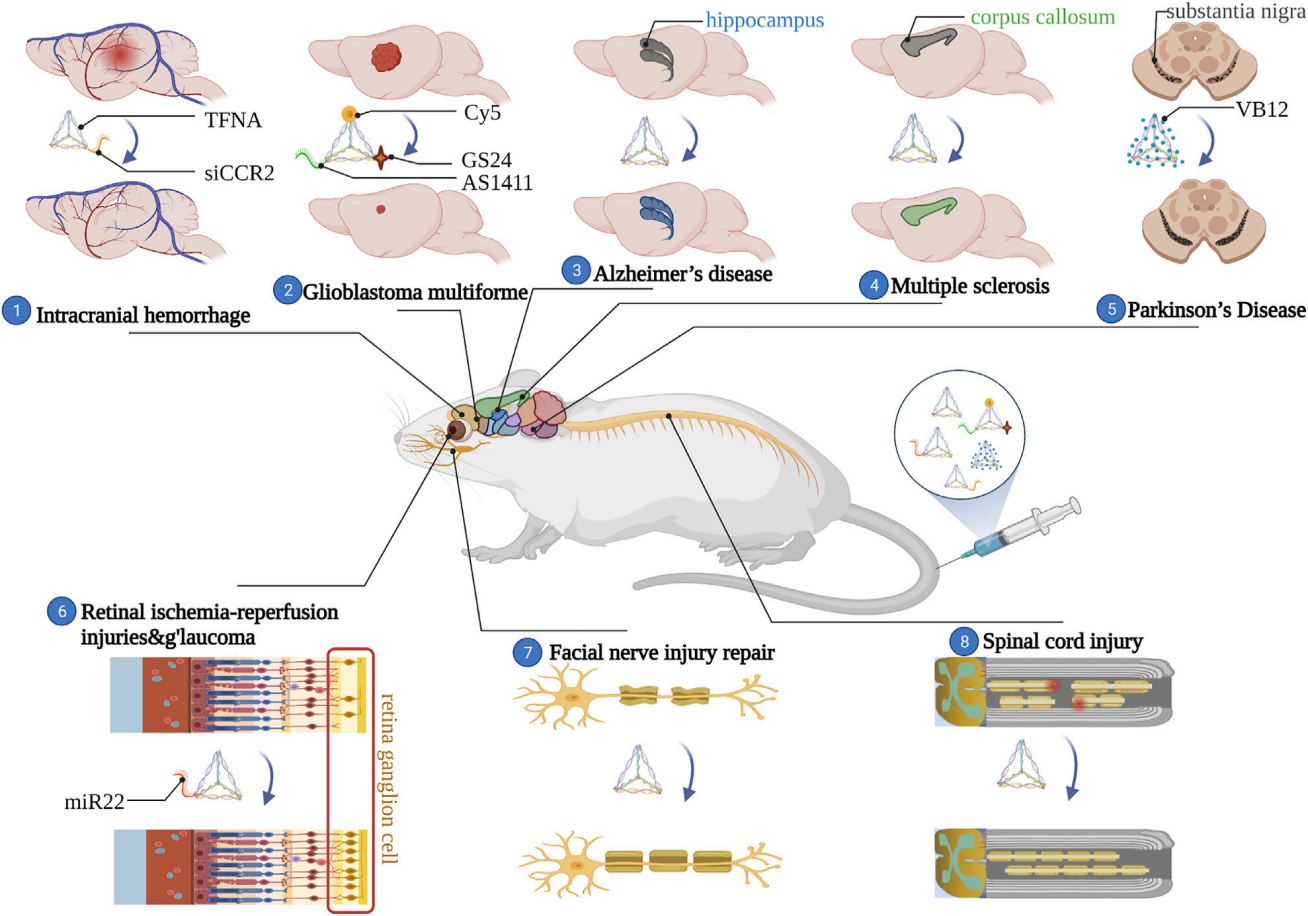
Application of tFNA-based nanomaterials in neurological disorders.

In conclusion, management of NDs is difficult due to poor understanding of the origin, the existence of BBB as well as the challenges in modifying existing drugs. Our analysis shows that the newly synthesized DNA nanomaterials, tFNA, are biocompatible, biodegradable and programmable with size/structure-controlled membrane permeability. During the past few years, surprising and exciting outcomes have been attained from several animal models of NDs such as PD, AD and GBM (Figure 4). Progress in the etiology or diagnosis of the NDs as well as development in the tFNA modification strategies would in turn enhance the application of tFNA in neuroscience. Besides, a wise and reasonable design of tFNA-based nanomaterials might bring great opportunities for the treatment of NDs. Conclusively, Zhang et al. detailly descripted four existing designing strategies of tFNA-based nanomaterials in biomedical treatment which we think could also be used in the treatment of NDs (Zhang et al., 2020b):

• Coat tFNA-based nanomaterials with protective agents such as cationic polymers to increase stability in the circulatory system and enhance release in nervous system;
• Append an aptamer such as GS24 to the vertex of tFNA to allow more drug to pass through the BBB in a shorter period of time;
• Direct incubation tFNA with small-molecular-weight drugs such as VB12 to increase solubility and utilization rate of drugs;
• Replace a short sequence of a single DNA strand by an antisense peptide nucleic acid to block the expression of unwanted genes in NDs.

While the promise that tFNA-based therapeutics hold is vast, the field of nanomedicine is still relatively new, with much to be learnt and explored. There are multiple challenges that are needed to be overcome in developing an ideal tFNA-based therapeutical system as we have mentioned above. But those yet unknown puzzles also push us to go deeper to unseal the underlying mysteries of nanotechnology.

## Abbreviations

tFNA, tetrahedral framework nucleic acid; NDs, neurological disorders; CNS, central nervous system; BBB, blood-brain barrier; Apt-TDN, aptamer-modified TDN; NGCs, neural stem cells; RGC, retinal ganglion cell; SCI, spinal cord injury; ICH, intracranial haemorrhage; CCR2, C-C chemokine receptor 2; AD, alzheimer’s disease; PD, parkinson’s disease; MPTP, 1-methyl-4-phenyl-1,2,3,6-tetrahydropyridine; VB12, vitamin B12; TVC, VB12-loaded tetrahedral framework nucleic acid; MS, multiple sclerosis; GBM, glioblastoma multiforme; GTG, gint4.T-tFNA-GMT8; TMZ, temozolomide; ROS, reactive oxygen species; MBP, myelin basic protein.
